# Birth preparedness, readiness planning and associated factors among mothers in Farta district, Ethiopia: a cross-sectional study

**DOI:** 10.1186/s12884-019-2325-4

**Published:** 2019-05-15

**Authors:** Miteku Andualem Limenih, Habitamu Gebrehana Belay, Habitamu Abie Tassew

**Affiliations:** 1 Department of Clinical Midwifery, School of Midwifery, College of Medicine and Health Sciences, University of Gondar, P.O. Box: 196, Gondar, Ethiopia; 2 Department of Midwifery, College of Health Sciences, Debretabor University, P.O. Box: 272, Debretabor, Ethiopia

**Keywords:** Birth preparedness, Complication readiness, Obstetric danger signs, Maternal mortality, Newborn health

## Abstract

**Background:**

Birth Preparedness and Complication Readiness (BP/CR) is the process of planning for normal birth and anticipating actions needed in case of emergency. Even though there is no adequate evidences on determinant factors, women and newborn need timely access to skilled care during pregnancy, childbirth, and the postpartum period. The aim of this study was to identify factors associated with the practice of birth preparedness and complication readiness plan among women who gave birth in the last 12 months in Farta District, Ethiopia, 2016.

**Method:**

A community-based cross-sectional study was conducted among 676 mothers from 1st October to December, 2016. Multistage sampling technique was used to select study participants. Data were collected using structured and pretested questionnaire. Bivariate and multivariable logistic regression models were fitted to identify factors associated with the practice of birth preparedness and complication readiness plan. An adjusted odds ratio with 95% confidence interval (CI) was computed to determine the level of significant.

**Result:**

The percentage of women implementing complication readiness plan and practicing birth preparedness was found to be 34%. Residence [Adjusted odds ratio (AOR): 5.94, 95% CI: 2.28–15.46)]; educational status [AOR: 2.87, 95% CI: (1.27–6.49)]; Antenatal care follow up [AOR: 3.67, 95% CI: (2.10–6.41)]; history of stillbirth [AOR: 3.05, 95CI: (1.20–7.78)]; knowledge of birth preparedness and complication readiness plans [AOR: 8.83, 95% CI: (5.01–15.58)]; knowledge of key danger signs during pregnancy [AOR: 3.91, 95% CI: (2.52–6.06)], *child birth* [AOR: 2.22, 95CI: (1.45–3.39)] and postpartum period [AOR: 1.99, 95% CI: (1.14–3.48)] were significantly associated with practice of birth preparedness and complication readiness plan.

**Conclusion:**

The overall proportion of women who prepared for birth and its complication readiness was found to be low. Educating women, encouraging pregnant women to utilize antenatal care, creating awareness on danger signs during pregnancy and childbirth might increase women’s birth preparation and complication readiness plan.

**Electronic supplementary material:**

The online version of this article (10.1186/s12884-019-2325-4) contains supplementary material, which is available to authorized users.

## Background

Globally, Maternal death due to complications during pregnancy and childbirth decreased by 50% from an estimated 523,000 in 1990 to 289,000 in 2013 [1]. Even though such progress is considerable, the average annual rate of decline is far below that is needed to be achieved by the Sustainable Development Goals (SDG) target 2030 of 5.5% [1].

High numbers of mothers are dying still in 2013, nearly 800 women died every day from obstetrical causes. Of this, low-income and middle-income countries alone account for 99% of maternal deaths [1, 2].

Ethiopia had made good progress toward improving maternal and child health. Despite this progress, big challenges are still within the health care system which is the basis of Ethiopia’s priorities including low utilization of maternal health services, such as skilled attendants at birth and high unmet need for family planning; limited availability of adolescent and youth sexual and reproductive health; lack of awareness of healthy behaviors; cultural barriers and inequities in health service utilization and quality of care [2].

In Ethiopia, up to 15% of mothers and newborns suffer serious complications that warrant referral to facilities providing comprehensive emergency obstetric and neonatal care service including caesarean sections, blood transfusion and emergency laparotomy [3].

Deliveries attended by skilled health personnel are one of the most important interventions to reduce maternal and child mortality [4, 5]. Rather than focusing on birth preparedness and complication readiness planning, in high-income countries, the focus is primarily on women’s psychological and physical comfort but in low-income and middle-income countries, there is a need of emphasis on birth preparation and potential complications [6].

Trends of maternal death showed Ethiopia is one of the five countries which have contributed to more than 50% of the world’s maternal death with 412 mothers per 100,000 live births in 2014. Most maternal deaths occur during delivery and postpartum period. The high maternal and neonatal mortality in the low – income and middle –income countries are due to three delays: delay in health seeking-behavior (delaying to seek medical care), delay in reaching a health facility and delay in getting the proper treatment. The possible causes of delays are logistic and financial constraints and lack of knowledge about maternal and neonatal health issues [7]. Therefore, the essential maternal and neonatal mortality reduction measures like; Emergency obstetric care, skilled birth attendant, postnatal care and arranging transportation in case of an emergency will reduce these delays [8].

In Ethiopian Health Centers and all level of Hospitals, women’s were provided information regarding to specific pregnancy complications during ANC care visits, including a severe headache, abdominal pain, vaginal bleeding, and leakage of vaginal fluid and blurred vision, but only half of the pregnant women receive the recommended minimum of four ANC care visits in developing countries [9]. One study in Ethiopian’s Hospital in 2011 revealed that the quality of care was below internationally accepted standards for ANC, labor and delivery service and essential new-born care which showed 40% of healthcare providers only were knowledgeable on how to prevent, identify and manage maternal complications [10].

Birth preparedness and complication readiness (BP/CR) is a process of planning for normal birth and anticipating the actions needed in case of an emergency and it is a comprehensive package designed to address delays by empowering women, her family, and the community to improve planning for birth and take actions in case of an emergency [6]. Components of birth preparedness and complication readiness plan includes; identifying a place of delivery, saving money, preparing essential items for childbirth, identifying a skilled provider, identifying a mode of transportation, arranging blood donors, arranging a way for communication, designating decision maker on her behalf, identifying emergency funds, being aware of the obstetric danger signs & the need to act immediately [11–13].

Studies in different regions of Ethiopia conducted among women in Robe district, Goba district, and Adigrat Town identified a very low magnitude of birth preparedness and complication readiness that is 16.5, 29.9 and 22.1% respectively [12–14]. Acquiring knowledge of key obstetric danger signs is the first essential step for women to identify factors and seek appropriate emergency care [15, 16]. However, there is limited evidence regarding factors associated with this low coverage in Ethiopia generally and in the study area particularly. Therefore, this study aimed to assess factors affecting birth preparedness and complication readiness plan among women who gave birth in the last 12 months in Farta district.

## Methods

### Study setting

A community-based cross-sectional study was used to determine the proportion of practice of birth preparedness and complication readiness plan among women who gave birth in the last 12 months in Farta District from October to December 2016 in Farta district. Farta district is one of the 15 districts in South Gondar Zone, situated in Amhara National Regional State, Ethiopia. It is 668 km far from the capital city of Ethiopia, Addis Ababa to the north direction. The district is subdivided into 41 rural and 2 urban kebeles (it is the smallest governmental administrative structural unit in Ethiopia but larger than a village). Based on the 2015 demographic survey, a total of 276,144 populations reside in the district, among these 139,923 are males and 136,221 are females and an estimated of 5047 women have a child less than 1 year of age [17]. In the district there are10 health centers, 56 health posts, and 4 private health clinics that are providing women and child health care services. The government of Ethiopia tried to promote the danger signs that can occur during pregnancy and *child birth* using community health extension worker in the region since 2010. But the extent of promoting the type of danger signs is limited since the health extension worker at the community level is not responsible to give the antenatal and delivery care services in the region which hinder to provide adequate information for the women.

### Operational definitions

There were multiple operational definitions related to knowledge and birth preparation in this current study.

A woman was considered as prepared for birth and its complication when she practiced or applied at least 6 items among 12 components of birth preparedness and complication readiness.

A woman was considered knowledgeable for birth preparedness and complication readiness when she mentioned at least 6 items of birth preparedness and complication readiness.

A woman was considered knowledgeable for danger signs of pregnancy when she mentioned at least three key danger signs of pregnancy.

A woman was considered knowledgeable for danger signs of labor and delivery when she mentioned at least three key danger signs of labor and delivery.

A woman was considered knowledgeable for danger signs of postpartum period when she mentioned at least three key danger signs of the postpartum.

### Inclusion and exclusion criteria

All women who gave birth in the last 12 months regardless of the birth outcome in the selected kebeles and who lived in the study area a minimum of 6 months were included. The exclusion criteria were women that were severely ill and unable to communicate during the study period.

### Sample size determination

Single population proportion formula was used to determine the sample size. 95% confidence interval (CI), a margin of error of 5 and 29.9% proportion of birth preparedness and complication readiness plan [12] was considered. We used a design effect of 2 to avoid the effect of the design that decreases the representativeness of the study. To compensate for non-response, 5% of the determined sample was added and the final sample size was 676.

### Sampling techniques

We used multistage sampling technique to select the sampling unit. First, all kebeles were stratified in to an urban and rural area. The district constitutes 41 rural and 2 urban kebeles. Simple random sampling technique was used to select one out of two urban kebeles and 16 out of 41 rural kebeles. Finally, the census was conducted at each selected kebele to register all women who have less than one-year age child and to create a sampling frame. The final calculated sample size was allocated proportionally to each selected kebele after the population size of each selected kebele was identified. The starting point from the sampling frame was obtained by using lottery methods then the study participants were selected from each kebele by using systematic random sampling techniques until the desired sample size was completed. When the eligible woman was not availed in household, the data collector went to their house repeatedly within the study period since the household is labeled and mapped before data collection rather than going to the next home to fulfill the sample size whereas, when there is more than one eligible woman in one household, the interviewed woman was selected via lottery methods.

### Data collection procedure and quality assurance

We used a pre-tested and structured questionnaire after literature review. The questionnaire (Additional file 1) was prepared in English, then translated to Amharic and back-translated to English by language experts to check its consistency. To assure the quality of the data, technical training was given before data collection for data collectors and also pre-testing was conducted on 10% of a sample size to pilot test the survey tool on kebeles outside the actual data collection site that have characteristics similar to the study population. Data collection was done by 17 trained diploma midwives and was supervised by five BSc midwives. Throughout the data collection period, the supervisor monitored the way of data collection and checked each filled questionnaire for its completeness. Finally, the data was cleaned after entry to ensure completeness.

### Data analysis and interpretation

The data were entered into a computer using Epidemiological Information (EPI-INFO) version 6 software and exported to SPSS (Statistical packages for social science) version 20. Descriptive statistics, binary and multivariable logistic regression analyses were used to identify associated factors. Variables having *P*-value ≤0.2 in the bivariate analysis were fitted into multiple logistic regression models to control the effect of confounding. Crude and adjusted odds ratio with their 95% CI were calculated to determine the strength and presence of association. A *p* value of ≤0.05 was considered to declare the level of significance.

The reference group of each independent variable had a value of 1, and the value for other groups was compared to that of the reference category. Odds ratio (OR) < 1 infers that persons in that category have a lower likelihood in practice of birth preparedness and complication readiness plan than persons in the reference category. Similarly, OR > 1 was designated increased probability of reporting as they had practised birth preparedness and complication readiness plan.

## Result

### Socio-demographic and obstetric characteristic of respondents, in Farta District, Northwest Ethiopia, 2016

A total of 676 mothers were interviewed with a response rate of 100%. 439(64.9%) mothers were in the age group of 20–34 with the mean age was (29.58 years + 6.12SD). 97% of respondents were married and 99% of mothers were an orthodox religious follower. Concerning to the educational status 363(53.7%) could not read and write and 51(7.5%) had attended grade 9 and above, and those who had at least once ANC follow - up visit were 536(78.8%) (Table 1).

**Table 1:** Socio-demographic and obstetric characteristic of respondents, in Farta District Northwest, Ethiopia, 2016 (*n* = 676)

Variable	Frequency	Percentage
Age		
< 20	72	10.7
20–34	439	64.9
> = 35	165	24.4
Marital status		
Currently in marital union	654	96.7
Currently not in marital union	22	3.3
Religion		
Orthodox	668	98.8
Muslim	6	0.9
Protestant	2	0.3
Residence		
Urban	29	4.3
Rural	647	95.7
Educational status		
Cannot read and write	363	53.7
Can read and wire	101	14.9
Elementary school (1–8)	161	23.8
Secondary school and above (Grade 9 and above)	51	7.5
Occupation		
House wife	626	92.6
Merchant	31	4.6
Employed	13	1.9
Others	6	0.9
Family size		
1–3	108	16.0
4–6	410	60.1
> = 7	158	23.4
Parity		
1	106	15.7
2–4	397	58.7
> = 5	173	25.6
ANC follow up		
Yes	532	78.7
No	144	21.3

### Respondents` awareness of birth preparedness and complication readiness

Five hundred forty-nine (81.2%) of respondents had heard about birth preparedness and complication readiness, of them 458(83.4%), 52(9.5%) and 39(7.1%) heard from their health care provider, family members and mass media respectively. Out of total respondents, 97(14.3%) were knowledgeable regarding birth preparedness and complication readiness. Commonly mentioned items of birth preparedness and complication readiness were; preparing essential items for clean and safe delivery 444(65.5%), identifying appropriate health facility 419(62%), making a plan for transportation 306(45.3%) and identifying the location of the nearest health facility where emergency service is provided 345(51%). Relatively the least mentioned items of birth preparedness and complication readiness were arranging blood donors in case of an emergency 5 (0.7%) and choosing a skilled provider for delivery 36(5.3%) (Table 2).

**Table 2:** Knowledge of women on birth preparedness and its complication readiness plan in Farta District, Northwest Ethiopia, 2016

	Variables(*n* = 676)	Frequency	Percent %
1	Identify appropriate health facility	419	62.0
2	Choose a skilled provider	36	5.3
3	Make a plan for transportation means	306	45.3
4	Make a plan for communication means	211	31.2
5	Save money to be used during emergency	287	42.5
6	Prepare essential items for clean and save delivery	444	65.5
7	Identify support people to help	115	17.0
8	Be able to identify the sign of an obstetric emergency	121	17.9
9	Know the importance of seeking care without delay when Complication occur	144	21.3
10	Have a plan to be able to respond immediately in the event of an emergency to avoid delays	188	27.8
11	Know the location of the nearest health facility where emergency service is provided	345	51.0
12	Arranging blood donors in case of an emergency	5	0.7

### Knowledge of respondents about key obstetric danger signs

**Fig. 1:**
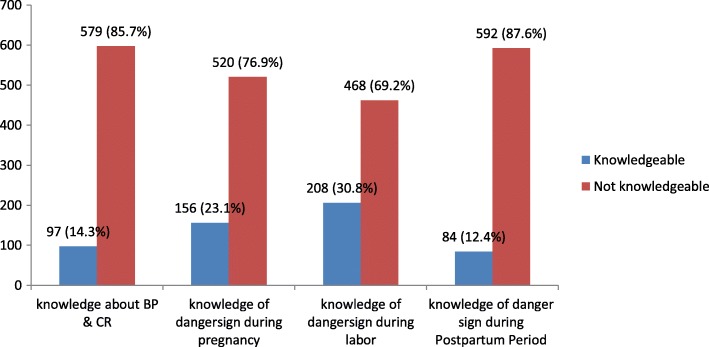
Knowledge of women on obstetric danger sign and birth preparedness and complication readiness plan among women who gave birth in the last 12 months in Farta district, Ethiopia, 2016

One hundred fifty-six (23.1%) were knowledgeable for obstetric danger signs that can be occurred during pregnancy, 208(30.8%) were knowledgeable for danger signs occurred during labor and delivery and 84(12.4%) were knowledgeable for danger signs occurred during the postpartum period (Fig. 1).

The most commonly mentioned items of danger signs during pregnancy were vaginal bleeding 276 (40.8%) and swollen hands and face 138(20.4%). During labor and delivery the majority of listed items was severe vaginal bleeding 436(51.2%) and prolonged labor > 12 h 280(41.4%). During the postnatal period, vaginal bleeding 486(71.9%) and severe headache 145(21.4%) were the most commonly mentioned items. The least mentioned items during pregnancy, childbirth and postpartum period were a loss of consciousness 57(8.6%), severe headache 46 (6.7%) and persistent fever 29 (4.3%) respectively (Table 3).

**Table 3:** Knowledge of respondents on danger signs during pregnancy, labour & delivery and post-partum period in Farta District, Northwest Ethiopia, 2016

I.Danger signs during pregnancy(*n* = 676)		Frequency	Percent%
1	Vaginal bleeding	276	40.8
2	Swollen hands and face	138	20.4
3	Blurred vision	121	17.9
4	High fever	89	13.2
5	Severe lower abdominal pain	111	16.4
6	Fits or loss of consciousness	57	8.6
II.Danger signs during labour and delivery (*n* = 676)			
1	Severe vaginal bleeding	436	51.2
2	Prolonged labour > 12 h	280	41.4
3	Hand, feet, cord or face appears first	211	31.2
4	Retained placenta	265	39.2
5	Fits or loss of consciousness	91	13.5
6	Severe headache	46	6.7
III.Danger signs during postnatal period (n = 676)			
1	Vaginal bleeding	486	71.9
2	Offensive Vaginal discharge	90	13.3
3	Severe Headache	145	21.4
4	Blurred vision	59	8.7
5	Fever	29	4.3
6	Fits or loss of consciousness	110	16.3

### The practice of respondents about preparation for birth and its complication

Of the total respondents, 58.3% identified appropriate health facility, 436(64.5%) make a plan for transportation means, 352((52.1) make a plan for communication means, 447(66.1%) prepare essential items for clean and safe delivery. Two hundred thirteen (31.5%) study participants saved money for an emergency situation. Signs of obstetric danger sign were identified by 122 (18%) study participants. Two hundred twenty-nine (33.9%) study participants reported that they were ready to avoid delay in response to emergency condition during labor and delivery. The least identified items were arranging blood donors in case of an emergency which was mentioned by 4(0.6%) study participants and choosing a skilled provider which was identified by 62(9.2%) study participants.

### Knowledge of women on birth preparedness and complication readiness plan

**Fig. 2:**
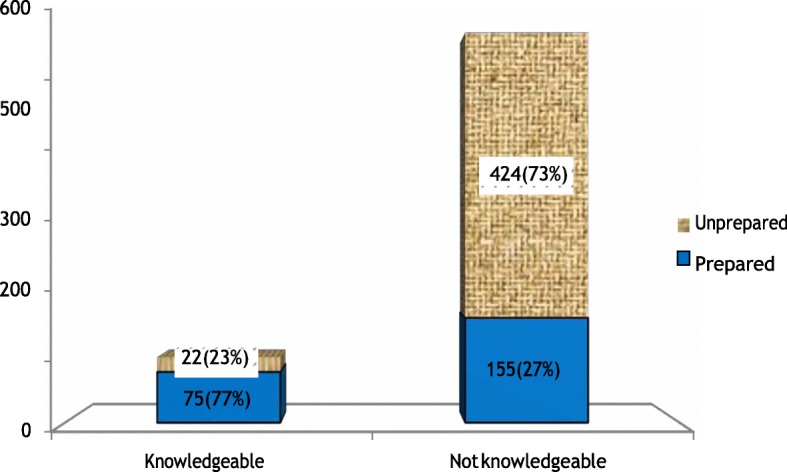
knowledge of women on birth preparedness and complication readiness practice among women who gave birth in the last 12 months in Farta district, Ethiopia, 2016

The proportion of women prepared for birth and its complication among those knowledgeable women about birth preparedness and its complication readiness were 75(77%), and among those who were not knowledgeable about birth preparedness and its complication readiness were 155 (27%) (Fig. 2).

### Factors associated with birth preparedness and complication readiness practice

Multivariable logistic regressions revealed that residence, educational status, ANC follow up visit, history of stillbirth, knowledge of birth preparedness and its complication readiness, knowledge of danger signs during pregnancy, childbirth and postpartum period were significantly associated with the practice of birth preparedness and complication readiness plan.

Women who were urban dweller were nearly six times more likely to prepare for birth and its complication as compared to their counterparts [AOR = 5.94, 95% CI = (2.28–15.46)]. Women who attended secondary school and above were nearly 3 times more likely to prepare for birth and its complication readiness as compared to women who are unable to read and write [AOR = 2.87, 95% CI = (1.27–6.49)]. Women who had a history of stillbirth were three times more likely to prepare for birth and its complication [AOR = 3.05, 95% CI = (1.20–7.78)]. Those women who had ANC follow-up visit were nearly four times more likely to prepare for birth and its complication [AOR = 3.67, 95%CI = (2.10–6.41)]. Those mothers who were knowledgeable for birth preparedness and complication readiness were nearly nine times more likely to prepare for birth and complication readiness compared to non-knowledgeable mothers [AOR = 8.83, 95% CI = (5.01–15.58)]. Mothers who were knowledgeable for danger signs during pregnancy, *child birth* and postpartum period were significantly associated with prepared for birth and its complication [AOR = 3.91 95% CI = (2.52–6.06)], [AOR =2.22, 95% CI = (1.45–3.39)] and [AOR =1.99, 95% CI = (1.14–3.48)] respectively (Table 4).

**Table 4:** Logistic regression analysis on factors associated with the practise of birth preparedness and complication readiness plan in Farta District, 2016

Variables(*N* = 676)	The practise of respondents on birth preparedness and its complication readiness		Crude OR(95% CI)	Adjusted OR(95% CI)
	Prepared (*n* = 230)	Not prepared (*n* = 446)		
Residence				
urban	20	9	4.62(2.07–10.33)	5.94(2.28–15.46)*
Rural	210	437	1	1
Educational status				
Cannot read and write	118	245	1	1
Can read and write	28	73	3.22(1.76–5.89)	2.67(1.24–5.75)*
Elementary education (Grade 1–8)	53	108	4.04(1.98–8.23)	2.48(1.04–5.94)*
Secondary Education (Grade 9–12) & above	31	20	3.16(1.65–6.06)	2.87(1.27–6.49)*
ANC follow-up visit				
Yes	206	326	3.16(1.97–5.06)	3.67(2.10–6.41)*
No	24	120	1	1
Parity				
1	41	65	1.05(0.64–1.73)	a
2–4	120	277	1.53(1.06–2.22)	a
≥ 5	69	104	1	1
History of Abortion				
Yes	10	7	2.85(1.07–7.59)	a
No	220	439	1	1
History of stillbirth				
Yes	17	11	3.16(1.45–6.86)	3.05(1.20–7.78)*
No	213	435	1	1
Knowledge of respondents for BP& CR plan				
Knowledgeable	75	22	9.33(5.60–15.52)	8.83(5.01–15.58)*
Not knowledgeable	155	424	1	1
Knowledge status of obstetric danger signs during pregnancy				
Knowledgeable	92	64	3.98(2.74–5.78)	3.91(2.52–6.06)*
Not knowledgeable	138	382	1	1
Knowledge status of obstetric danger signs during *child birth*				
Knowledgeable	104	104	2.71(1.93–3.81)	2.22(1.45–3.39)*
Not knowledgeable	126	342	1	1
Knowledge status of obstetric danger signs during the postnatal period				
Knowledgeable	44	40	2.40(1.51–3.81)	1.99(1.14–3.48)*
Not knowledgeable	186	406	1	1

*with italicized value indicated that a statistically significant association at 95% Confidence interval (CI) that did not include 1 in the interval

1 = reference category

^a^not significant in stepwise backward logistic regression. Hosmer and Lemanshow test for multivariable log reg. = 0.85

## Discussion

This study revealed that the practice of birth preparedness and complication readiness plan was found to be 34%. This is in line with studies done in Goba district that used the same definition for birth preparedness and complication readiness, Southern Ethiopia and Jimma Zone that used the more likely comparable definition for birth preparedness and complication readiness [12, 18, 19]. The level of birth preparedness and its complication readiness was found to be low as compared to the studies in Tanzania and Nigeria that used the nearly comparable definition for BP & CR [20, 21]. This could be due to different socio-demographic characteristics and different health care system implementation. The finding of this study was found to be higher than that of studies done in Adigrat town, Arsi zone and Southern Ethiopia that used a nearly similar definition for BP & CR [13, 14, 18]. The possible reason for this variation might be the study period and the sample size variation.

The study revealed that 21.4% of respondents delivered their last child at a health institution. About eight in every 10 women (79%) received at least once ANC follow-up visit in their last pregnancy. The finding is consistent with the Ethiopian Demographic and Health Survey (EDHS) 2016 result which found that 62% of women who gave birth in the last 5 years received at least once ANC follow-up care and 20% of rural women and 79% of urban women gave birth at a health facility [22]. This finding is also in line with the result of the study done in a northern Nigerian Community in which 21.9% of the respondent delivered their last child in a health institution and 75.5% of the participants had at least one ANC visit [23]. Antenatal care from skilled provides is important to monitor pregnancy and reduce morbidity and mortality risks from mother and child during pregnancy, delivery and postpartum period.

Knowledge on birth preparedness and key obstetric danger signs in pregnancy, *child birth* and postpartum period were significantly associated with the practice of birth preparedness and its complication readiness. Women who were knowledgeable for birth preparedness and its complication readiness were 8.83 times more likely prepared for birth and its complication [AOR = 8.83, 95% CI = (5.01–15.58)] as compared to their counterparts and also those who were knowledgeable for obstetric danger signs of pregnancy [AOR = 3.91, 95% CI = (2.52–6.06)], *child birth* [AOR = 2.22, 95% CI = (1.45-3.39)] and postpartum period [AOR = 1.99, 95% CI = (1.14–3.48)] were more likely prepared for birth and its complication. This is in line with the studies done in Arsi, southern Ethiopia, sub Saharan Africa and Tanzania [14, 23–25]

Other studies revealed that knowledge of obstetric danger signs during pregnancy and postpartum period was not associated with birth preparedness and complication readiness [13, 18, 19] and knowledge of obstetric danger signs during *child birth* was not associated with the practice of birth preparedness and complication readiness [12]. But it is not supported in the finding of this study. The possible reason might be socio-demographic variation, variation in the definition of knowledgeable and the methodology variation.

Women with secondary education and above were 2.87 times more likely to be prepared for birth and its complication as compared to women who were unable to read and write [AOR = 2.87, 95% CI = (1.27–6.49)]. Mothers who have attended ANC follow-up visit in their last pregnancy were 3.67 times more likely prepared for birth and its complication than those who had no ANC follow up visit [AOR = 3.67, 95% CI = (2.10–6.41)]. This finding is in line with the study done in Goba district, Oromia region; Adigrat town, northern Ethiopia and Robe woreda, Arsi zone [12–14]. This might be due to ANC follow up might provide an opportunity for the women to be counseled about birth preparedness and its complication readiness and key obstetric danger signs.

Women who were urban dwellers were 5.94 times more likely prepared for birth and its complication as compared to rural counter parts [AOR =5.94, 95% CI = (2.28–15.46)]. This is consistent with the studies done in Goba district, and Sub- Saharan Africa [12, 19]. This might be due to variation in access to information, education, accessibility and availability of maternal health service.

Among the obstetric factors, mothers who had a history of *still birth* were prepared for birth and its complication than mother who did not have a history of *still birth* [AOR = 3.05, 95% CI = (1.20–7.78)]. This is similarly reported in Adigrat town, northern Ethiopia [13]. This could be due to mothers who had fear of past history of birth complications tending to attend ANC follow up visit and give birth in the health institutions.

A significant proportion of respondents were not knowledgeable about birth preparedness and its complication readiness. This finding is in line with other previous studies [12–14]. Among 12 spontaneously asked components of birth preparedness and complication readiness items, more than half of respondents identified appropriate health facility, planned for transportation, planned for communication means and prepared essential items for *child birth*. This is consistent with the studies done in Adigrat town and Robe woreda [13, 14]. Majority of respondents mentioned vaginal bleeding as a major obstetric danger sign during pregnancy (40.8%), *child birth* (51.2%) and the postpartum period (71.9%). This is consistent with the studies done in Tsegedie District, Aleta Wondo District and Mapawa district [15, 26, 27]. Women having knowledge of obstetric danger signs might have a chance to improve their decision-making ability on their maternal and child health care service utilization and can respond immediately in the event of emergencies to avoid delays. Low awareness may be a cause of failure to recognize the sign of obstetric emergency and delaying to respond immediately in the event of an emergency [16].

The study revealed that a small number of women were well prepared for birth and its complication. Therefore Ministry of health maternal and child health directorate, Amhara regional health bureau and Farta district health office are strongly recommended to work hard to improve birth preparedness and its complication readiness of women.

Farta district health office in collaboration with the district educational office should further strengthen their activity to empower women with education. Furthermore, the regional policy makers and program planners are recommended to increase educational access to the women which will make alert them in preparing to birth and complication. This might be due to, as the level of education of increase, women empowerment would increase which might increase birth preparedness and complication readiness plan [7, 15, 28].

The Farta district health officers and the health care providers are recommended to increase awareness about obstetric danger sign, increase quality and accessibility of antenatal care services for every woman at the community and providing adequate information about the risk of lack of birth preparedness through community mobilization during maternal conference, during ANC follow up visit and postnatal follow up visit. This might be due to the fact that women awareness on ANC, and when they got quality of maternal health care service, their practice on birth preparedness and complication readiness plan might increase [16, 19].

## Limitation of study

Monthly income might be one of the causes that can affect the practice of birth preparedness and complication readiness plan but in this study, the assessment of their revenues throughout data collection was not precise so that it was not reflected during the analysis. Even though we tried to minimize, still there might be recall bias and social desirability bias. In addition, the district surveyed in this study may also not be representative of other districts in Ethiopia or regions beyond Ethiopia.

## Conclusion

The proportion of birth preparation and complication readiness practice among women who gave birth in the last 12 months was found to be low. Knowledge statuses of respondents about birth preparedness and its complication readiness and on key obstetric danger signs were very poor. Knowledge of key obstetric danger signs during pregnancy, delivery and postnatal period, knowledge about preparation for birth and its complication, ANC follow up visit, residence, history of *still birth*, and educational status were factors significantly associated with birth preparedness and complication readiness practice.

## Additional file

Additional file 1: English version questionnaire: English language questionnaire that used to assess Birth preparedness, readiness planning and associated factors among mothers in Farta district, Ethiopia. (DOCX 25 kb)

## Abbreviations

ANC: Antenatal Care
AOR: Adjusted Odds Ratio
CI: Confidence Interval
COR: Crud Odds Ratio
EDHS: Ethiopian Demography and Health Survey
SDG: Sustainable Development Goals
WHO: World Health Organization
