# Supplementary material for: Birth preparedness, readiness planning and associated factors among mothers in Farta district, Ethiopia: a cross-sectional study
Source: BMC Pregnancy Childbirth. 2019 May 15;19:171. doi: 10.1186/s12884-019-2325-4 (PMC6521428; doi:10.1186/s12884-019-2325-4)
Supplement: Supplementary file 1 — English version questionnaire: English language questionnaire that used to assess Birth preparedness, readiness planning and associated factors among mothers in Farta district, Ethiopia. (DOCX 25 kb) [file 12884_2019_2325_MOESM1_ESM.docx]

**English questionnaire**

**Title: Birth preparedness, readiness planning and associated factors among mothers in Farta district, Ethiopia: a cross-sectional study**

**Part one: Maternal characteristic**

**Table one: Socio- Demographic and economic characteristics of the respondents inFarta Woreda, South Gondar Zone, Ethiopia, 2016**

| S. No | Question | Response | Code |
| --- | --- | --- | --- |
| 101 | Currently age in years? | -------------------------in year |  |
| 102 | What is your place of residence? | 1. Urban 2. Rural |  |
| 103 | What is your religion? | 1. Orthodox 2. Muslim 3. Protestant 4. If other specify…………………….. |  |
| 104 | To which ethnic group do you belong? | 1. Amhara 2. Tigray 3. Oromo 4. If other specify…………………….. |  |
| 105 | What is your current maritalstatus? | 1. currently in marital union 2. currently not in marital union |  |
| 106 | What is your level of education? | 1. cannot read and write 2. Can read and write 3. Primary (1-8th class) 4. Secondary and above ( >12^th^ grade ) |  |
| 107 | What is your Occupation? | 1. House wife 2. Merchant 3. Employee 4. Daily labourer 5. Farming 6. If other specify……………….. |  |
| 108 | What is your partner level of education? | 1. cannot read and write 2. Can read and write 3. Primary (1-8th class) 4. Secondary and above ( >12th grade ) |  |
| 109 | What is your partner Occupation? | 1. Farming 2. Governmental Employee 3. Non-Governmental Employee 4. Merchant 5. Daily laborer 6. if other Specify-------------- |  |
| 110 | How much distances do you travel to the nearby health facility? | 1. In kilometre by car……………… 2. Inhours by foot…………………….. |  |
| 111 | How much your family monthly income? | …………………in birr |  |
| 112 | How many your family sizes? | ------------------------------ |  |

## Section 2: Obstetrics and Gynecology factors influencing birth preparedness and complication readiness plan, Farta Woreda, South Gondar Zone, Ethiopia, 2016

| 201 | Number of Gravida | ……………..in number |  |
| --- | --- | --- | --- |
| 202 | Number of parity | ……………..in number |  |
| 203 | Have you ever experienced miscarriage/abortion before 28 weeks of gestational age | 1. yes 2. no |  |
| 204 | Have you ever history of still birth | 1. yes 2. no |  |
| 205 | Have you had ANC follow-up visit in your last pregnancy? | 1. yes 2. no | If Ans. no ,skip to 301 |
| 206 | If yes to Q 205 --- how many times you follow? | ------------------------ |  |
| 207 | Are you advised to prepare for birth and its complication during your ANC follow up time? | 1. yes 2. no |  |
| 208 | Where you gave your last birth? | 1. Home 2. Health institution |  |
| 209 | Have you been experienced past obstetric complication before your last birth? | 1. Yes 2. No |  |

## Section 3:- Knowledge of respondents about birth preparedness and complication readiness plan,Farta Woreda, South Gondar Zone, Ethiopia, 2016

| 301 | What do you know about components of preparation forbirth and its complication readiness?  **( possible to select more than one answer)** | 1. Identify appropriate health facility with skilled provider for delivery 2. Choose a skilled provider 3. Make a plan for transportation means and a person to accompany to identify health facility on the start of labor 4. Make a plan for communication means 5. Save money to be used during emergency 6. Prepare essential items for clean and save delivery 7. Identify support people to help 8. Be able to identify sign of obstetric emergency 9. Know importance of seeking care without delay when complications occur 10. Have a plan to be able to respond immediately in the event of emergency to avoid delays 11. Know the location of the nearest health facility where emergency service is provided. 12. Arranging blood donors in case of an emergency |  |
| --- | --- | --- | --- |
| 302 | When do you think obstetric danger signs occur?  **( possible to select more than one answer)** | 1. ……… during pregnancy 2. ……….During labor and delivery 3. ………During post-partum period 4. ………..unawareness |  |
| 303 | What types of obstetric danger signs occur during pregnancy? | 1. Vaginal bleeding 2. Swollen hands and face 3. Blurred vision 4. High fever 5. Severe lower abdominal pain 6. Fits or loss of consciousness |  |
| 304 | What types of Obstetric danger signs can occur during labour and deliver?  **( possible to select more than one answer)** | 1. Severe vaginal bleeding 2. Prolonged labour > 12 hours 3. Hand, feet, cord or face appears first 4. Retained placenta 5. Fits or loss of consciousness 6. Severe headache |  |
| 305 | What types of dangersigns can occur during post-partum period?  **( possible to select more than one answer)** | 1. Vaginal bleeding 2. Offensive Vaginal discharge 3. Severe Headache 4. Blurred vision 5. Fever 6. Fits or loss of consciousness |  |

## Section 4:-practice of respondents about birth preparedness and complication readiness plan,Farta Woreda, South Gondar Zone, Ethiopia, 2016

| 401 | Identify appropriate health facility with skilled provider for delivery | 1. Yes 2. No |  |
| --- | --- | --- | --- |
| 402 | Choose a skilled provider | 1. Yes 2. No |  |
| 403 | Make a plan for transportation means and a person to accompany to identify health facility on the start of labor | 1. Yes 2. No |  |
| 404 | Make a plan for communication means | 1. Yes 2. No |  |
| 405 | Save money to be used during emergency | 1. Yes 2. No |  |
| 406 | Prepare essential items for clean and save delivery | 1. Yes 2. No |  |
| 407 | Identify support people to help | 1. Yes 2. No |  |
| 408 | Be able to identify sign of obstetric emergency | 1. Yes 2. No |  |
| 409 | Know importance of seeking care without delay when complications occur | 1. Yes 2. No |  |
| 410 | Have a plan to be able to respond immediately in the event of emergency to avoid delays | 1. Yes 2. No |  |
| 411 | Know the location of the nearest health facility where emergency service is provided. | 1. Yes 2. No |  |
| 412 | Arranging blood donors in case of an emergency | 1. Yes 2. No |  |
